# Supplementary material for: Elevated FBXO45 promotes liver tumorigenesis through enhancing IGF2BP1 ubiquitination and subsequent PLK1 upregulation
Source: eLife. 2021 Nov 15;10:e70715. doi: 10.7554/eLife.70715 (PMC8641947; doi:10.7554/eLife.70715)
Supplement: Supplementary file 1. [file elife-70715-supp1.docx]

**Supplementary file 1. Univariate and multivariate analyses indicating associations between overall survival and various risk factors in 105 HCC patients**

| **Variables** |  | **OS** | |
| --- | --- | --- | --- |
|  | N | Hazard ratio (95% Cl)* | P value |
| **Univariables** |  |  |  |
| FBXO45(high vs. low) | (57 vs. 48) | 2.641(1.644-4.242) | **<0.001***** |
| Age(>55y vs. ≤55y) | (29 vs. 76) | 0.782(0.465-1.314) | 0.353 |
| Gender(male vs. female) | (89 vs. 16) | 1.207(0.637-2.286) | 0.564 |
| Histologic grade(G1G2 vs. G3) | (87 vs. 18) | 2.247(1.303-3.875) | **0.004**** |
| TNM Stage(I VS. II III IV) | (35 vs. 70) | 2.029(1.214-3.392) | **0.007**** |
| Tumor size (>5cm vs. <=5cm) | (82 vs. 23) | 1.603(0.897-2.864) | 0.111 |
| Recurrence(Present vs. Absent) | (24 vs. 81) | 1.795(0.938-3.435) | 0.077 |
| Metastasis(Present vs. Absent) | (53 vs. 52) | 2.523(1.577-4.035) | **<0.001***** |
| **Multivariables** |  |  |  |
| FBXO45(high vs. low) | (57 vs. 48) | 2.447(1.490-4.021) | **<0.001***** |
| Histologic grade (G1G2 vs. G3) | (87 vs. 18) | 1.960(1.108-3.467) | **0.021*** |
| TNM Stage(I VS. II III IV) | (35 vs. 70) | 0.630(0.281-1.412) | 0.262 |
| Metastasis(Present vs. Absent) | (53 vs. 52) | 3.005(1.485-6.079) | **0.002**** |

*Calculated using cox proportional hazards regression*

**P ≤ 0.05; **P ≤ 0.01; ***P ≤ 0.001 were considered statistically significant.*
