# Supplementary material for: Elevated FBXO45 promotes liver tumorigenesis through enhancing IGF2BP1 ubiquitination and subsequent PLK1 upregulation
Source: eLife. 2021 Nov 15;10:e70715. doi: 10.7554/eLife.70715 (PMC8641947; doi:10.7554/eLife.70715)
Supplement: Supplementary file 2. [file elife-70715-supp2.docx]

**Supplementary file 2. Relationships between *FBXO45* mRNA expression and clinicopathologic characteristics in 253 HCC patients**

| Variables | Cases | *FBXO45*  High level | *FBXO45*  Low  level | p Value |
| --- | --- | --- | --- | --- |
| Age(years) |  |  |  | 0.365 |
| ≤55 | 91 | 51 | 40 |  |
| >55 | 162 | 81 | 81 |  |
| Gender |  |  |  | 0.734 |
| Female | 81 | 41 | 40 |  |
| Male | 172 | 91 | 81 |  |
| Hepatitis B |  |  |  | 0.073 |
| Present | 96 | 57 | 39 |  |
| Absent | 157 | 75 | 82 |  |
| AFP |  |  |  | **0.011*** |
| ≤200 | 184 | 87 | 97 |  |
| >200 | 69 | 45 | 24 |  |
| Histologic Grade |  |  |  | **<0.0001***** |
| G1G2 | 147 | 61 | 86 |  |
| G3G4 | 106 | 71 | 35 |  |
| Tumor stage |  |  |  | **0.01*** |
| T1 | 144 | 65 | 79 |  |
| T2 T3 T4 | 109 | 67 | 42 |  |
| TNM Stage |  |  |  | **0.007**** |
| I | 143 | 64 | 79 |  |
| II-IV | 110 | 68 | 42 |  |

*Calculated using the χ 2 test.*

**P ≤ 0.05 ; **P ≤ 0.01; ***P ≤ 0.001 were considered statistically significant.*
