# Supplementary material for: Elevated FBXO45 promotes liver tumorigenesis through enhancing IGF2BP1 ubiquitination and subsequent PLK1 upregulation
Source: eLife. 2021 Nov 15;10:e70715. doi: 10.7554/eLife.70715 (PMC8641947; doi:10.7554/eLife.70715)
Supplement: Supplementary file 3. [file elife-70715-supp3.docx]

**Supplementary file 3. Univariate and multivariate analyses indicating the associations between overall survival and various risk factors in 253 HCC patients**

| Variables |  | OS | |
| --- | --- | --- | --- |
|  | N | Hazard ratio (95% Cl)* | P value |
| **Univariables** |  |  |  |
| *FBXO45*(high vs. low) | (132 vs. 121) | 1.656(1.039-2.640) | **0.034*** |
| Age(>55y vs. ≤55y) | (162 vs. 91) | 1.659(1.003-2.746) | **0.049*** |
| Gender(male vs. female) | (172 vs. 81) | 0.622(0.393-0.984) | **0.042*** |
| HBV(negative vs. positive) | (157vs. 96) | 2.179(1.292-3.674) | **0.003**** |
| Serum AFP(>200ng/ml vs. <=200ng/ml) | (69 vs. 184) | 1.051(0.638-1.733) | 0.844 |
| Histologic grade(G1G2 vs. G3G4) | (147 vs. 106) | 1.481(0.939-2.337) | 0.092 |
| TNM Stage(I VS. II III IV) | (143 vs. 110) | 1.718(1.090-2.709) | **0.020*** |
| T(T1 vs. T2 T3 T4) | (144 vs. 109) | 1.660(1.054-2.615) | **0.029*** |
| N(N0 vs. N1 NX) | (186 vs. 67) | 1.661(1.010-2.733) | **0.046*** |
| M(M0 vs. M1 MX) | (191 vs. 62) | 2.268(1.403-3.664) | **0.001***** |
| **Multivariables** |  |  |  |
| *FBXO45*(high vs. low) | (132 vs. 121) | 1.673(1.020-2.741) | **0.041*** |
| Age(>55y vs. <=55y) | (162 vs. 91) | 1.380(0.804-2.369) | 0.243 |
| Gender(male vs. female) | (172 vs. 81) | 0.809(0.491-1.334) | 0.406 |
| HBV(negative vs. positive) | (157vs. 96) | 1.496(0.805-2.779) | 0.203 |
| TNM Stage(I VS. II III IV) | (143 vs. 110) | 3.077(0.364-25.981) | 0.302 |
| T(T1 vs. T2 T3 T4) | (144 vs. 109) | 0.471(0.058-3.834) | 0.482 |
| N(N0 vs. N1 NX) | (186 vs. 67) | 0.773(0.415-1.440) | 0.417 |
| M(M0 vs. M1 MX) | (191 vs. 62) | 2.587(1.418-4.719) | **0.002**** |

*Calculated using the cox proportional hazards regression.*

*P ≤ 0.05; **P ≤ 0.01; ***P ≤ 0.001 were considered statistically significant.*
