# Supplementary material for: Elevated FBXO45 promotes liver tumorigenesis through enhancing IGF2BP1 ubiquitination and subsequent PLK1 upregulation
Source: eLife. 2021 Nov 15;10:e70715. doi: 10.7554/eLife.70715 (PMC8641947; doi:10.7554/eLife.70715)
Supplement: Supplementary file 4. [file elife-70715-supp4.docx]

**Supplementary file 4. FBXO45-interacting proteins identified by Co IP-MS**

| Accession | Protein ID |
| --- | --- |
| Q13885 | TUBB2A |
| P0C2W1 | FBXO45 |
| P63208 | SKP1 |
| P08107 | HSPA1A |
| Q8NCL4 | GALNT6 |
| P07814 | EPRS |
| O00571 | DDX3X |
| P57723 | PCBP4 |
| Q9BQA1 | WDR77 |
| Q9NZB2 | FAM120A |
| Q96BM9 | ARL8A |
| P47914 | RPL29 |
| P31943 | HNRNPH1 |
| Q9UMD9 | COL17A1 |
| P61006 | RAB8A |
| P49368 | CCT3 |
| Q9NX62 | IMPAD1 |
| Q13045 | FLII |
| Q9H5X1 | FAM96A |
| Q86W92 | PPFIBP1 |
| P11586 | MTHFD1 |
| Q15418 | RPS6KA1 |
| Q14568 | HSP90AA2 |
| Q9NZI8 | IGF2BP1 |
| P50990 | CCT8 |
| Q14527 | HLTF |
| Q92734 | TFG |
| P55265 | ADAR |
| P37198 | NUP62 |
| Q5M775 | CYTSB |
| P01616 | KV203 |
| P02795 | MT2A |
| O14744 | PRMT5 |
| Q99805 | TM9SF2 |
| Q6PK04 | CCDC137 |
| P61019 | RAB2A |
| P62988 | RPS27A |
| Q9Y6H1 | CHCHD2 |
| P62937 | PPIA |
| Q9Y333 | LSM2 |
| Q14596 | NBR1 |
| Q9Y6B6 | SAR1B |
| P55060 | CSE1L |
| P82094 | TMF1 |
| P27449 | ATP6V0C |
| A6ZKI3 | FAM127A |
