# Supplementary material for: Elevated FBXO45 promotes liver tumorigenesis through enhancing IGF2BP1 ubiquitination and subsequent PLK1 upregulation
Source: eLife. 2021 Nov 15;10:e70715. doi: 10.7554/eLife.70715 (PMC8641947; doi:10.7554/eLife.70715)
Supplement: Supplementary file 5. [file elife-70715-supp5.docx]

**Supplementary file 5. Relationships between the IGF2BP1 protein and clinicopathological characteristics in 105 HCC patients**

| Variables | Cases | IGF2BP1 | IGF2BP1 | p Value |
| --- | --- | --- | --- | --- |
|  |  | High level | Low level |  |
| Age(years) |  |  |  | 0.280 |
| ≤55 | 76 | 53 | 23 |  |
| >55 | 29 | 17 | 12 |  |
| Gender |  |  |  | 0.442 |
| Female | 16 | 12 | 4 |  |
| Male | 89 | 58 | 31 |  |
| TNM Stage |  |  |  | **0.019*** |
| I | 35 | 18 | 17 |  |
| II-IV | 70 | 52 | 18 |  |
| Histologic Grade |  |  |  | **0.006**** |
| G1G2 | 87 | 53 | 34 |  |
| G3 | 18 | 17 | 1 |  |
| Tumor size |  |  |  | **0.030*** |
| ≤5cm | 23 | 11 | 12 |  |
| >5cm | 82 | 59 | 23 |  |
| Recurrence |  |  |  | 1.000 |
| Present | 24 | 16 | 8 |  |
| Absent | 81 | 54 | 27 |  |
| Metastasis |  |  |  | 0.073 |
| Present | 53 | 31 | 22 |  |
| Absent | 52 | 39 | 13 |  |

*Calculated using the χ 2 test.*

**P ≤ 0.05; **P ≤ 0.01 were considered statistically significant.*
